# Collagen turnover profiles in chronic kidney disease

**DOI:** 10.1038/s41598-019-51905-3

**Published:** 2019-11-05

**Authors:** Daniel Guldager Kring Rasmussen, Lene Boesby, Signe Holm Nielsen, Martin Tepel, Sophie Birot, Morten Asser Karsdal, Anne-Lise Kamper, Federica Genovese

**Affiliations:** 1 Nordic Bioscience, Herlev, Denmark; 2 Department of Cardiovascular and Renal Research, Institute of Molecular Medicine, University of Southern Denmark, Odense, Denmark; 3 Department of Medicine, University Hospital Roskilde, Roskilde, Denmark; 4 Department of Nephrology, Herlev Hospital, Herlev, Denmark; 5 Department of Biotechnology and Biomedicine, Technical University of Denmark, Kgs, Lyngby, Denmark; 6 Department of Nephrology, Odense University Hospital, Odense, Denmark; 7 Department of Nephrology, Rigshospitalet, Copenhagen, Denmark

**Keywords:** Diagnostic markers, Diagnostic markers, Kidney diseases

## Abstract

Renal fibrosis is a hallmark of chronic kidney disease (CKD) caused by an imbalance between formation and degradation of extracellular matrix proteins. We investigated the collagen turnover profile of 81 non-dialysis CKD stage 2–5 patients by measuring peptides reflecting formation and degradation of collagen type (COL) I, III, IV, and VI. Based on the collagen turnover profile, we identified four clusters of patients. Cluster 1 contained one patient with prostate cancer, who had a distinct collagen turnover. The other clusters generally had severe (Cluster 2), moderate (Cluster 4), or mild CKD (Cluster 3). Cluster 4 patients were characterized by higher levels of COL III, COL IV, and COL VI (all p < 0.001) degradation fragments in plasma, while patients in Clusters 2 and 4 had higher levels of COL VI formation (p < 0.05). COL IV fragments in plasma were lower in Cluster 2 (p < 0.01). Urinary COL III fragments decreased from Cluster 3 to 4, and from Cluster 4 to 2 (both p < 0.001). We show that patients with similar kidney function have a different collagen remodeling profile, suggesting that different phenotypes exist with different disease activity and potentially disease progression. Biomarkers of collagen remodeling could provide additional information to traditional markers of renal function.

## Introduction

Chronic kidney disease (CKD) may be caused by different etiologies and the prevalence in the adult population is estimated to be between 8–16%^1–3^. CKD is characterized by a progressive reduction of glomerular filtration rate (GFR). The loss in GFR is mainly due to the destruction of the functional filtration surface area, a process driven by renal fibrosis^4–6^. Various studies have shown that an increased collagen deposition in the interstitial matrix and glomerular basement membrane is found in CKD^7–9^. However, in humans it is unknown whether increased formation and reduced degradation promotes these changes, because of the lack of adequate tools to distinguish these processes.

Biomarkers reflecting structural changes in the renal extracellular matrix (ECM) may reflect disease activity and could be useful tools in the development of drugs that inhibit or delay the progression of fibrosis. Such biomarkers could be used: (i) to reflect changes to the tissue due to a specific treatment, (ii) as surrogate endpoints in clinical trials, and (iii) to enrich clinical trials for patients with a high disease activity (i.e. fast progressors), that are more likely to encounter a clinically relevant outcome in the time of the trial and benefit the most from the treatment^10^.

The most abundant collagens in the interstitial matrix of the kidney are collagen type I (COL I) and III (COL III), while collagen type IV (COL IV) is the main component of the glomerular basement membrane^10^. Recent discoveries on the up-regulation of collagen type VI (COL VI) in renal fibrosis have promoted interest in this collagen, which is found in the interface between the interstitial matrix and basement membrane^11^. In renal fibrosis, these collagens are upregulated and the activity of the proteases responsible for their remodeling is altered^9,10,12,13^. Biomarkers of collagen remodeling could be used to delineate the pathological alterations to the ECM during CKD progression, thereby increasing our understanding of CKD. We measured biomarkers of collagen turnover to investigate the hypothesis that different profiles of ECM remodeling could differentiate CKD patients regardless of their kidney function.

We measured: a fragment of COL VI released during deposition in the ECM (PRO-C6^14,15^) and a marker of MMP-2 mediated COL VI degradation (C6M^13^); markers reflecting COL IV turnover (PRO-C4^16,17^, C4M^18^ and C4Ma3^18^); markers for COL III formation (PRO-C3^19–21^) and degradation mediated by ADAMTS-4 (C3A), cathepsins B, L, S and K (C3C^22^) and MMP-9 (C3M^23–25^); markers reflecting COL I formation (PRO-C1^26^) and MMP-2, 9, and 13-mediated degradation (C1M^27^).

We used principal component analysis to identify clusters of patients with distinct differences in collagen turnover and characterized the clinical profile of the selected clusters.

## Results

### Patient characteristics

The clinical characteristics of the 81 patients enrolled in the current study are presented in Table 1. Median [interquartile range (IQR)] age of the study participants was 62 [52–73] years and body mass index (BMI) was 27.2 [25.1–29.8] kg/m^2^. There were 24 (29.6%) females and 20 patients (24.7%) had diabetes mellitus. Systolic blood pressure was 125 [116–140], and diastolic blood pressure was 78 [70–80]. Median eGFR was 33 [16–60] ml/min/1.73 m^2^, and albuminuria was 89 [26–644] mg/g protein. The cohort consisted of 20 patients with CKD stage 2, 21 patients with CKD stage 3, 22 patients with CKD stage 4, and 18 patients with CKD stage 5.

**Table 1:** Patient characteristics.

Variable	Summary (n = 81)
Age (years)	62 [52–73]
Female, n (%)	24 (29.6)
Diabetes, n (%)	20 (24.7)
BMI (kg/m^2^)	27.2 [25.1–29.8]
Systolic BP (mmHg)	125 [116–140]
Diastolic BP (mmHg)	78 [70–80]
PP (mmHg)	50 [38–61]
eGFR	33 [16–60]
UACR (mg/g)	89 [26–644]
hsCRP (mg/L)	1.8 [1.1–3.9]
Hemoglobin (mM)	8.1 [7.3–8.9]
p-triglycerides (mM)	1.4 [1.1–2.2]
p-LDL (mM)	2.8 [2.0–3.5]
p-HDL (mM)	1.3 [1.0–1.7]
AIx	29 [21–36]
AIx/75	22 [14–29]
aPWV	9.1 [7.8–11.1]

Data are presented as median interquartile range for continuous variables, and n (%) for categorical variables. Abbreviations: BMI, body mass index; BP, blood pressure; PP, pulse pressure; eGFR, estimated glomerular filtration rate; UACR, urinary albumin:creatinine ratio; hsCRP, high sensitivity C-reactive protein; p, plasma; LDL, low density lipoprotein; HDL, high density lipoprotein; AIx, augmentation index; AIx/75, AIx per 75 heartbeats per minute; aPWV, aortic pulse wave velocity.

### Cluster-generation based on collagen turnover profile

Despite the underlying etiology, all patients with chronic kidney disease develop interstitial fibrosis^4–6^. To assess the turnover of collagens in the renal ECM we quantified fragments of collagens reflecting both degradation, deposition, and formation (Table 2).

**Table 2:** Characteristics of assays used to assess collagen turnover.

Biomarker	Specifications	Applicable in	Technical References
COL I ASSAYS	**Interstitial matrix**		
C1M	Recognizes a fragment of the α1 chain of COL I generated by MMP-2, 9 and 13	Plasma (P-C1M) Urine (U-C1M)	^27^
PRO-C1	Recognizes an internal epitope in the N-terminal pro-peptide of COL I	Plasma (P-PRO-C1)	^26^
COL III ASSAYS	**Interstitial Matrix**		
C3A	Degradation fragment of COL III generated by ADAMTS-4	Plasma (P-C3A)	
C3C	Degradation fragment of COL III generated by cathepsin B, L, S and K	Plasma (P-C3C) Urine (U-C3C)	^22^
C3M	Degradation fragment of COL III generated by MMP-9	Plasma (P-C3M) Urine (U-C3M)	^23– 25^
sauC3M	Degradation fragment of COL III (sandwich ELISA) generated by MMP-9	Urine (U-sauC3M)	
PRO-C3	Recognizes the cleavage site of the N-terminal pro-peptide of COL III	Plasma (P-PRO-C3) Urine (U-PRO-C3)	^19– 21^
COL VI ASSAYS	**Interface between interstitial matrix and basement membrane**		
C6M	Degradation product of the α1 chain of COL VI generated by MMP-2	Plasma (P-C6M)	^33, 34^
PRO-C6	Recognizes the C-terminal of α3 chain in COL VI which is released upon deposition in the ECM	Plasma (P-PRO-C6) Urine (U-PRO-C6)	^28, 29^
COL IV ASSAYS	**Glomerular basement membrane**		
C4M	Degradation fragment of the α1 chain of COL IV generated by MMP-12	Plasma (P-C4M) Urine (U-C4M)	^18^
C4Ma3	Degradation fragment of the α3 chain of COL IV generated by MMP-12	Plasma (P-C4Ma3)	^18^
PRO-C4	Internal epitope in the 7S domain of type IV collagen	Plasma (P-PRO-C4)	^16, 17^

**Figure 1:**
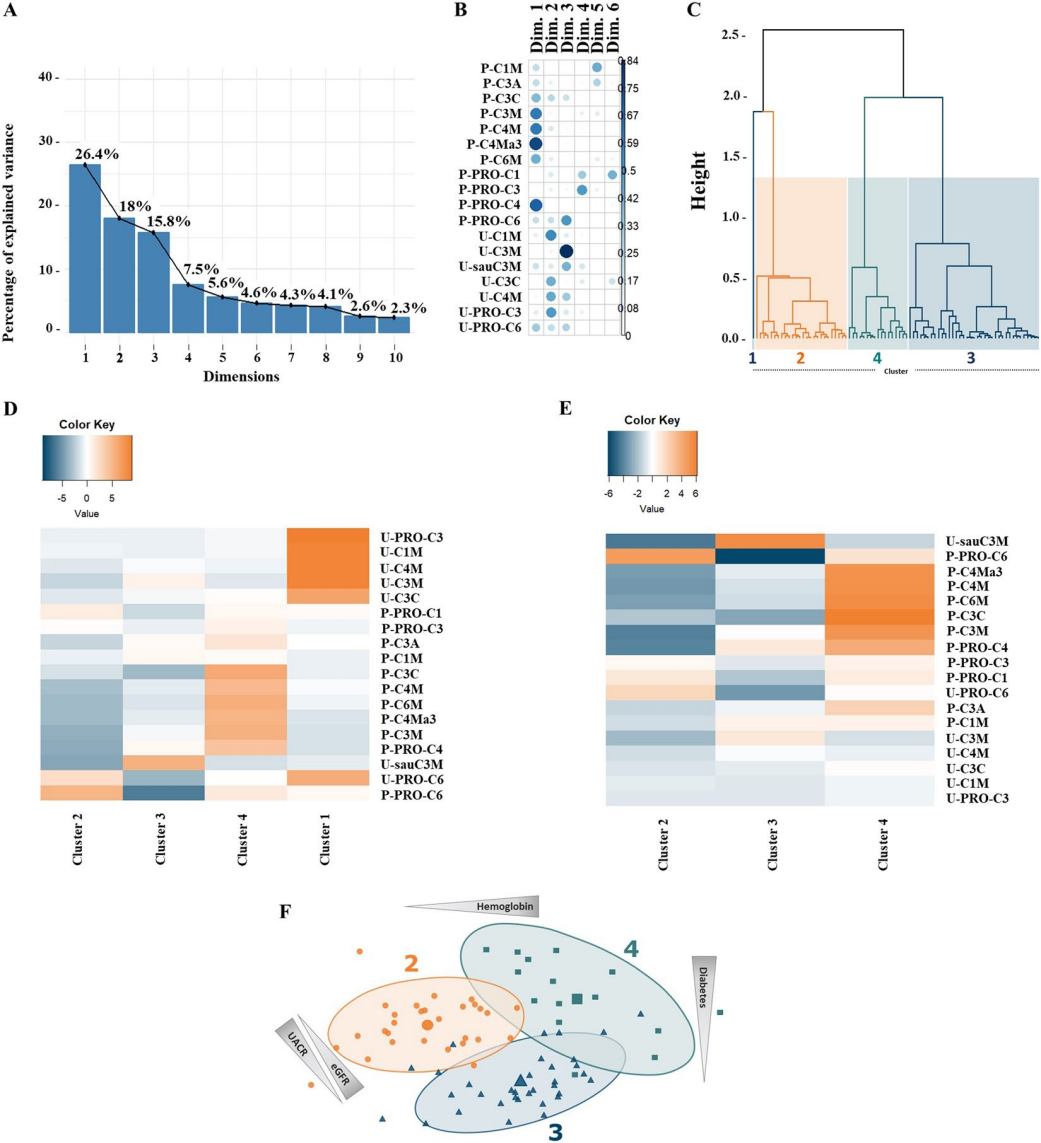
Cluster generation based on collagen turnover. The biomarker values were log-transformed in order to give similar weights by reducing the influence of extreme values or outliers (i.e. reducing absolute variation) and to linearize relationships between variables. To remove features with low variance and to generate a more robust clustering in subsequent steps, we applied principle component analysis on the log-transformed biomarker values. (**A**) We selected 6 dimensions for subsequent analysis, as these 6 dimensions accounted for approximately 80% of the overall variance. (**B**) We then depicted the relative contribution of each of the measured collagen biomarkers in the 6 dimensions. (**C**) In an unbiased clustering analysis it was determined that 4 clusters was optimal. (**D**) The collagen biomarker abundance in the four clusters, in comparison to the overall population, was visualized with heatmaps. This was done by using the test statistics of the ANOVA, which allowed the measurement of the extremeness of the values on the same scale. (**E**) As Cluster 1 only contained 1 patient with prostate cancer, we removed this cluster to highlight differences in collagen turnover biomarkers of Clusters 2–4. (**F**) Characterization of the 3 predominant clusters (Cluster 2–4). Differences in clinical characteristics between clusters were visualized. Actual differences are presented in Table 3.

Clusters were generated based on fragments of collagen turnover found both in plasma and urine. In the principal component analysis, the number of dimensions that best determined the overall variance in the cohort was 6 dimensions (Fig. 1A). We then depicted the relative contribution of each of the measured collagen biomarkers in the 6 dimensions (Fig. 1B). If two biomarkers are found in the same dimension (i.e. on the same vector), this means that they have the same direction in space and are thus to some extent correlated. Dimension 1 predominantly contained plasma markers of COL III degradation mediated by cathepsins (P-C3C) and MMP-9 (P-C3M), and COL IV degradation (P-PRO-C4, P-C4M and P-C4Ma3). Dimension 2 primarily contained urinary markers of COL I degradation (U-C1M), cathepsin mediated COL III degradation (U-C3C), COL IV degradation (U-C4M), and COL III formation (U-PRO-C3). Dimension 3 contained the urinary markers of COL III generated by MMP-9 (U-C3M and U-sauC3M), the plasma and urinary markers of COL VI formation (P-PRO-C6, U-PRO-C6), and the urinary COL IV degradation marker (U-C4M). Dimension 4 mainly contained formation markers of COL I (P-PRO-C1) and COL III (P-PRO-C3) in plasma. Dimension 5 contained the degradation marker of COL I in plasma (P-C1M), and the marker of COL III degradation generated by ADAMTS-4 (P-C3A). Lastly, dimension 6 mainly contained the plasma fragment of COL I formation (P-PRO-C1).

Based on the collagen biomarkers, unbiased clustering analysis revealed 4 clusters of patients (Fig. 1C). As Cluster 1 contained only one patient, we looked into the medical records of the patient and found that the patient was diagnosed with prostate cancer. This, together with the overt nephropathy and advanced CKD (Table 3), may explain the marked difference in fragments of collagen turnover of this patient (Fig. 1D). The patient in Cluster 1 had markedly higher level of markers of urinary COL I (U-C1M), COL III (U-C3M and U-C3C), and COL IV (U-C4M) degradation, and COL III (U-PRO-C3) and COL VI (U-PRO-C6) formation. To look at biomarkers of collagen turnover in Clusters 2–4, we did a heatmap excluding Cluster 1 (Fig. 1E).

**Table 3:** Cluster characteristics.

Variable	Cluster 1 (n = 1)	Cluster 2 (n = 26)	Cluster 3 (n = 37)	Cluster 4 (n = 17)	P-value
Age (years)	78 {-)	65 [57–74]	58 [50–69]	65 [54–73]	0.10
Female, n (%)	0 (0)	6 (23.1)	14 (37.8)	4 (23.5)	0.48
Diabetes, n (%)	1 (100)	10 (38.5)	3 (8.1)	6 (35.3)	**0**.**007**
BMI (kg/m^2^)	26.3 [-]	28.8 [24.1–31.5]	27.2 [25.8–28.9]	25.7 [23.6–29.0]	0.58
Systolic BP (mmHg)	135 [-]	125 [120–142]	126 [115–140]	118 [112–129]	0.29
Diastolic BP (mmHg)	80 [-]	76 [70–80]	80 [75–84]	70 [70–78]	**0**.**02**
PP (mmHg)	55 [-]	53 [45–64]	45 [36–56]	50 [36–60]	0.28
eGFR (ml/min/1.73 m^2^)	10 [-]	15 [14–20]	62 [43–75]	28 [14–42]	**<0**.**0001**
UACR (mg/g)	38700 [-]	430 [88–1389]	37 [14–138]	89 [33–571]	**0**.**002**
hsCRP (mg/L)	3.2 [-]	1.9 [1.3–3.2]	1.6 [1.0–3.3]	2.9 [1.4–4.7]	0.59
Hemoglobin (mM)	8.5 [-]	7.1 [6.6–7.8]	8.7 [7.7–9.2]	8.5 [7.8–8.8]	**0**.**0007**
p-triglycerides (mM)	1.8 [-]	1.3 [1.1–2.0]	1.6 [1.1–2.1]	1.5 [1.0–3.0]	0.89
p-LDL (mM)	2.2 [-]	2.6 [1.7–3.5]	3.0 [2.5–3.7]	2.3 [1.7–2.9]	0.08
p-HDL (mM)	1.5 [-]	1.2 [1.0–1.5]	1.4 [1.2–1.7]	1.3 [0.9–1.7]	0.39
AIx	40 [-]	27 [25–35]	30 [21–36]	25 [14–37]	0.53
AIx/75	41 [-]	22 [16–28]	23 [18–29]	18 [8–31]	0.29
aPWV	19.7 [-]	9.1 [8.1–10.7]	8.8 [7.4–11.1]	9.6 [7.8–10.8]	0.27

Data are presented as median interquartile range for continuous variables, and n (%) for categorical variables. Abbreviations: BMI, body mass index; BP, blood pressure; PP, pulse pressure; eGFR, estimated glomerular filtration rate; UACR, urinary albumin:creatinine ratio; hsCRP, high sensitivity C-reactive protein; p, plasma; LDL, low density lipoprotein; HDL, high density lipoprotein; AIx, augmentation index; AIx/75, AIx per 75 heartbeats per minute; aPWV, aortic pulse wave velocity. Differences between clusters were assessed with a Kruskal Wallis test for continuous variables, and a Chi-Squared test for categorical variables.

We investigated whether the different clusters presented differences in terms of clinical variables (Table 3 and Fig. 1F). There were less diabetic patients in Cluster 3 compared to the other groups (p = 0.007). Diastolic blood pressure was slightly higher in Cluster 3 than in Cluster 4 (p = 0.02). We found that Cluster 3 had significantly higher eGFR than all other clusters (p < 0.0001). Moreover, Cluster 4 had significantly higher eGFR than Cluster 2 (p = 0.04). Cluster 3 had significantly lower UACR than Cluster 2 (p = 0.002). Cluster 2 had significantly lower levels of hemoglobin than patients in the other clusters (p = 0.0007). Based on eGFR, UACR, and hemoglobin, the identified clusters contained patients with apparently mild CKD (Cluster 3), moderate CKD (Cluster 4), and advanced CKD (Cluster 2). Cluster 3 contained patients with high eGFR, low UACR, and normal hemoglobin levels; Cluster 4 contained patients with moderately reduced eGFR, moderately increased UACR, and normal hemoglobin levels; and Cluster 2 contained patients with low eGFR, high UACR, and low hemoglobin levels.

**Figure 2:**
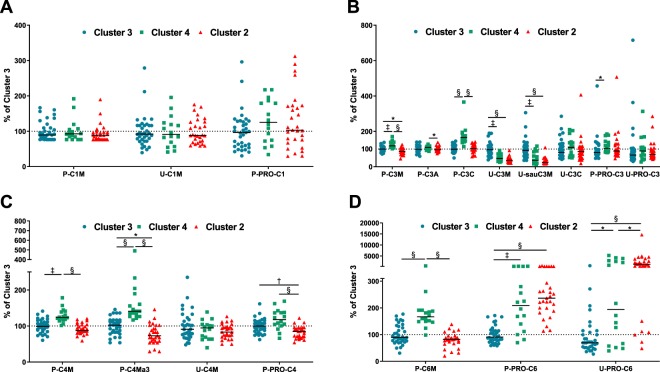
Collagen turnover biomarkers in Clusters 2–4.Collagen fragments reflecting degradation and formation processes of (**A**) COL I, (**B**) COL III, (**C**) COL IV, and (**D**) COL VI were shown for patients in Cluster 2–4. Data are presented as percentage of the average biomarker levels in Cluster 3 and depicted as scatter plot with median. Differences between groups was assessed by a Kruskal-Wallis test with Dunn’s multiple comparisons test. Significance level: *p < 0.05, ^†^p < 0.01, ^‡^p < 0.001, ^§^p < 0.0001.

Generation of Clusters 2, 3, and 4 seemed to be mainly driven by fragments of collagens found in plasma (Fig. 1E). No differences in markers of COL I turnover were seen between the clusters (Fig. 2A). Urinary levels of COL III degradation markers (U-C3M and U-sauC3M) were lower in Cluster 4 than in Cluster 3 (both p < 0.001; Fig. 2B) and were even lower in Cluster 2 (both p < 0.0001; Fig. 2B). In plasma, markers of COL III degradation (P-C3M and P-C3C) were higher in Cluster 4 than both Clusters 2 and 3 (all p < 0.001). The marker of COL III formation (P-PRO-C3) was significantly higher in Cluster 4 than in Cluster 3 (p = 0.02). In plasma, the degradation marker of the α1 chain of COL IV (P-C4M) was elevated in Cluster 4 compared to Clusters 2 and 3 (both p < 0.001), but no differences in the level of the urinary COL IV degradation marker (U-C4M) were seen (Fig. 2C). The degradation marker of the α3 chain of COL IV (P-C4Ma3) was significantly higher in Cluster 4 compared to Clusters 2 and 3 (both p < 0.0001) and was also higher in Cluster 3 compared to Cluster 2 (p < 0.05; Fig. 2C). The marker of COL IV degradation in plasma (P-PRO-C4) was significantly higher in Clusters 3 and 4 compared to Cluster 2 (both p < 0.01; Fig. 2C). The COL VI degradation marker in plasma (P-C6M) was significantly higher in Cluster 4 compared to both Cluster 2 and 3 (both p < 0.0001; Fig. 2D). The marker of COL VI formation in both plasma (P-PRO-C6) and urine (U-PRO-C6) was significantly lower in Cluster 3 compared to Cluster 2 and 4 (all p < 0.05; Fig. 2D). Moreover, the urinary marker of COL VI formation (U-PRO-C6) was significantly higher in Cluster 2 than in Cluster 4 (p < 0.05; Fig. 2D).

**Figure 3:**
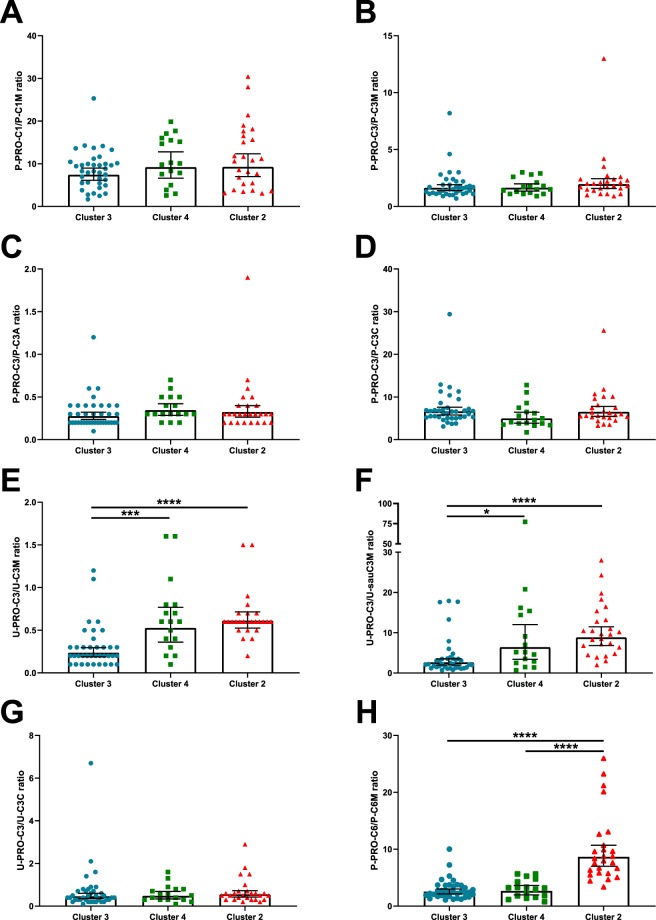
Ratios between collagen formation and degradation. Formation/degradation ratio of COL I ((**A**) P-PRO-C1/P-C1M), COL III ((**B**) P-PRO-C3/P-C3M, (**C**) P-PRO-C3/P-C3A, (**D**) P-PRO-C3/P-C3C, (**E**) U-PRO-C3/U-C3M, (**F**) U-PRO-C3/U-sauC3M, (**G**) U-PRO-C3/U-C3C), and COL VI ((**H**) P-PRO-C6/P-C6M). Results are shown as geometric mean with 95% confidence intervals. Differences between groups was assessed by a Kruskal-Wallis test with Dunn’s multiple comparisons test. Significance level: *p < 0.05, **p < 0.01, ***p < 0.001, ****p < 0.0001.

The balance between degradation and formation of collagens was investigated by calculating the ratio between fragments of formation and degradation for COL I, III, and VI where biomarker values were available in the same sample matrix (Fig. 3). The patient in Cluster 1 was excluded from the analysis. As P-PRO-C4 is likely to reflect degradation, rather than formation, we did not perform the ratio between this biomarker and P-C4M and P-C4Ma3. No difference in P-PRO-C1/P-C1M (Fig. 3A), P-PRO-C3/P-C3M (Fig. 3B), P-PRO-C3/P-C3A (Fig. 3C), P-PRO-C3/P-C3C (Fig. 3D) were observed between Clusters 2–4 for plasma. The mean [95% CI] of U-PRO-C3/U-C3M (Fig. 3E) was significantly lower in Cluster 3 compared to Cluster 4 (0.300 [0.22–0.38] vs. 0.66 [0.43–0.88], p = 0.0004), and Cluster 2 (0.300 [0.22–0.38] vs. 0.66 [0.54–0.77], p < 0.0001)(Fig. 3E). The mean [95% CI] U-PRO-C3/U-sauC3M ratio was significantly lower in Cluster 3 compared to Cluster 4 (4.03 [2.46–5.61] vs. 12.85 [3.33–22.38], p = 0.014), and Cluster 2 (4.03 [2.46–5.61] vs. 10.65 [8.02–13.27], p < 0.0001)(Fig. 3F). No difference in mean U-PRO-C3/U-C3C (Fig. 3G) was observed between the clusters. The mean [95% CI] P-PRO-C6/P-C6M ratio was significantly increased in Cluster 2 compared to both Cluster 3 (9.99 [7.55–12.44 vs. 2.91 [2.32–3.49], p < 0.0001) and Cluster 4 (9.99 [7.55–12.44 vs. 3.09 [2.27–3.91], p < 0.0001)(Fig. 3H).

Furthermore, we investigated whether the collagen turnover biomarkers were significantly different between patients stratified by CKD stage (Supplementary Table 1). We found that some of the biomarkers were significantly different in patients stratified by CKD stages; U-C3M, U-sauC3M, and P-PRO-C4 declined (all p < 0.01), and U-PRO-C3, P-PRO-C6, and U-PRO-C6 increased with CKD stage (all p < 0.05).

## Discussion

In the current study, previously described markers reflecting formation of COL I (PRO-C1^26^), III (PRO-C3^19–21^), and VI (PRO-C6^28–32^) and degradation products of COL I (C1M^27^), III (C3A, C3C^22^, and C3M^23–25^), IV (PRO-C4^16,17^, C4M^18^ and C4Ma3^18^) and VI (C6M^33,34^) were applied to identify and characterize clusters of CKD patients with differences in turnover of the main collagens found in the renal ECM. We identified different collagen turnover profiles in patients with CKD, which may highlight a shift in collagen turnover with progression of disease. Interestingly, many of the degradation fragments that were significantly different between the identified clusters were not significantly different between patients stratified by CKD stage. The collagen markers may be used to increase the understanding of pathological collagen turnover during disease progression.

Collagens are the main structural component of the fibrotic tissue, and biomarkers of collagen formation and degradation may provide valuable insights on the state of the tissue. As an imbalance of collagen turnover favoring increased deposition of collagens and fibrosis progression are closely linked^35,36^, biomarkers of collagen turnover may be able to identify patients with fast progression of CKD^9,10^. In the current study the main collagen turnover clusters (Clusters 2–4) seemed to preferentially contain patients with mild CKD (Cluster 3), moderate CKD (Cluster 4), and advanced CKD (Cluster 2).

During deposition of COL VI in the matrix, a fragment of the α3 chain is released, and may thus be used as a measure for COL VI formation^14,15,28^. As PRO-C6 recognizes the released fragment, and PRO-C6 was significantly elevated in patients of Clusters 4 and 2 with moderate and advanced CKD, respectively, our present findings are in line with reports showing COL VI accumulation in fibrotic kidneys^37,38^. Moreover, elevation of COL VI degradation (P-C6M^33,34^) was only observed in Cluster 4, and not in patients from Cluster 2 with advanced disease. When looking at the ratio of the P-PRO-C6 and P-C6M biomarkers as an indicator of COL VI turnover, we saw that only Cluster 2 had a significantly increased formation/degradation ratio. The increase of P-C6M in Cluster 4 may therefore be a compensatory mechanism to degrade accumulating COL VI. This may be indicative of a shift in COL VI turnover that favors a substantial increase in COL VI deposition with increasing disease severity. The decreased COL VI degradation may in part be due to reduced protease activity^12^ and in part to an increased cross-linking of collagen fibers that render them resistant to proteolytic processes. It is increasingly accepted that collagens are not merely structural proteins, but rather active participants in tissue turnover through interaction with both their immediate surroundings and distant sites through the release of active fragments called matrikines^36,39,40^. Several experimental data indicate a pathophysiological relevance of COL VI for progressive kidney disease. During development of the kidney, COL VI is highly expressed, but seems to be replaced during organ maturation^41^. In a study by Nerlich and co-authors it was shown that COL VI was progressively upregulated from diffuse to nodular glomerulosclerosis in kidneys from patients with diabetes^37^. Valerie Groma showed a marked increase in COL VI staining in CKD patients with progressive fibrosis, and that these areas had a high presence of myofibroblasts^38^. COL VI is located in the interface between the interstitial matrix and the glomerular basement membrane where it controls the organization of the matrix and cell orientation^42,43^. Findings by our group indicate that PRO-C6 in urine is independently associated with progression of CKD^30^, and levels in plasma were independently associated with mortality in CKD patients^29^. Moreover, we recently showed that levels of PRO-C6 in patients with type 1 and 2 diabetes were independently associated with all-cause mortality, cardiovascular events, and deterioration of kidney function, and added incremental predictive value to traditional risk factors for predicting these outcomes^31,32^. The concomitant increase in both plasma and urinary PRO-C6 indicates that the accumulation in plasma is not merely due to a reduced filtration, as seen for other biomarkers, such as plasma creatinine. Collectively, this supports the involvement of COL VI in progressive CKD and supports our findings of increased PRO-C6 in clusters of patients with moderate to advanced CKD. Even though collagens are generally considered to provide tensile strength to tissue, the stiffness of the resulting collagen fibers differs. COL VI forms a characteristic and distinctive network of beaded microfilaments in the ECM where it amongst others facilitates correct cell-orientation, and organization of the ECM^11^. The importance of COL VI in the interface between the interstitial matrix and the basement membrane is seen in the lung where COL VI provides elasticity and mechanical support^44^. Absence of COL VI in mice led to an altered basement membrane structure, and a decrease in cell–ECM interactions, which affects pulmonary elasticity, and a reduced exercise tolerance^45^. COL VI deficiency leads to abnormal fibrillogenesis, with an accompanying decrease in functionality of the tissue and stiffness, indicating that COL VI contributes to the maintenance of the mechanical properties of the tissue^46^. Furthermore, COL VI directly binds to cell-surface receptors such as NG2, thereby activating the Akt–GSK-3β–β-catenin–TCF–LEF axis^47^, the phosphoinositide 3-kinase (PI3K) pathway^48^, and induces epithelial–mesenchymal transition^49^. A fragment released by COL VI upon deposition in the ECM, namely endotrophin, increased the cell proliferation and angiogenesis^49^. As PRO-C6 detects this fragment, levels of this biomarker may aid in selecting patients that are more likely to have an unfavorable outcome due to having ongoing processes of inflammation, fibrosis, and apoptosis. These patients may be more likely to respond to a given treatment. This may ultimately aid the physician, and clinical trials by enrichment for patients with active disease.

The most abundant group of collagens in connective tissue are COL I and III. The presence of both COL I and III are believed to provide a tissue with high tensile strength, but also contribute, together with elastin, to the extensile properties of elastic arteries and the aorta^50,51^. The tensile strength is partially achieved by cross-linking which does not only occur between the chains of one collagen type, but rather between collagens, such as seen for cross-links between COL I and III^52^. Interestingly, in experiments investigating the tensile strength of different ratios of COL I and III, it was shown that an increased ratio of COL III versus COL I in the tissue may soften the tissue – as compared to a tissue with predominantly COL I^53^. Also, it has been shown that COL IIII is markedly upregulated in tissues with ongoing wound-healing^54^. Previous studies focusing on histological alterations of the kidney during renal disease have shown that COL III increases in fibrotic kidneys^55^. In plasma, we observed an increase in COL III degradation fragments in patients with moderate disease (Cluster 4) compared to patients with mild (Cluster 3) and advanced CKD (Cluster 2). In contrast, levels of U-C3M and U-sauC3M gradually decreased from Cluster 3 to 4, and from 4 to 2. With progression of CKD, MMP-9 activity has been shown to decrease^12^. As the investigated COL III degradation fragments in urine are generated by MMP-9, a reduced activity would cause a progressive decline in fragments containing this neo-epitope. In support of our findings, various proteomic studies have shown decreased COL I and III fragments in urine of CKD patients compared to healthy controls^56–60^. Previous findings by our group showed that levels of urinary C3M is lower in patients with IgA nephropathy with increasing CKD stage^25^. Combined with the findings of this study, this suggests that the decrease is a general phenomenon in patients with kidney disease, not linked to a specific underlying etiology. As the pattern of C3M in urine and plasma differs, we speculate that U-C3M could reflect a reduced degradation of COL III specifically in the kidney, whereas P-C3M may reflect systemic changes. If the U-C3M fragments were merely a result of an altered filtration and permeability, we would expect increased U-C3M levels, and gradually decreasing levels of P-C3M. Furthermore, no correlation was observed between U-C3M and P-C3M. If the reduced levels of urinary COL III fragments (U-C3M and U-sauC3M) reflect a local decrease in COL III degradation of the kidney, the clinician may use this to monitor the effect of treatment on the ability to remove accumulating COL III, or to enroll patients for clinical trials that investigate the effect of potential anti-fibrotic treatments on COL III turnover.

Our findings revealed a marked difference in COL IV turnover in CKD patients. The degradation markers of the α1 and α3 chain of COL IV increased in Cluster 4 compared to the other clusters. The marker P-PRO-C4, shown to be correlated to the amount of COL IV and total collagen in rats^16^, decreased significantly in Cluster 2 (advanced CKD patients). Nerlich and co-authors showed a reduced staining for COL IV with a concomitant increase in COL VI during nodular glomerulosclerosis in patients with diabetes^37^. This may partially explain the decrease in the COL IV markers and the concomitant increase in the marker of COL VI formation (PRO-C6) that was observed in our study for patients in clusters with more advanced disease.

Interestingly, many of the collagen degradation fragments that were significantly altered between the identified collagen turnover clusters were not significantly different when patients were stratified based on CKD stages. This may indicate that the identification of patients based on biomarkers of collagen turnover may identify clusters of patients with active disease, and that we are not just stratifying patients based on a static measurement of kidney function.

The strength of the present study is the use of an array of novel, robust assays for assessment of collagen formation and degradation in humans. A limitation of the current study is the cross-sectional design and a relatively small patient number. Because we measured samples from an observational study, we can only hypothesize on any causal relationships between the biomarkers and CKD pathogenesis. Another limitation is that not all assays were applicable for use in both plasma and urine and it is thus not possible to completely characterize the presence of these fragments in both matrices. Finally, the measured collagens are not unique for the renal tissue. Other comorbidities could thus affect the levels of the individual biomarker. However, as histologic studies have shown elevated levels of the investigated collagens in kidney disease, it is likely that the findings, at least partially, reflect alterations to the kidney.

In conclusion, we showed that assessment of collagen turnover can identify subgroups of patients with different collagen turnover and disease severity. Markers of collagen degradation were higher in patients with moderate disease and were lower in advanced disease. Interestingly, only patients in Cluster 2 with advanced disease had an increased COL VI formation/degradation ratio. The markers may be applied and investigated in prospective CKD cohorts to delineate pathological collagen turnover during disease progression. The findings could have wider implications for assigning clinical classification and predicting outcomes, but that is beyond the scope of this work.

## Methods

### Patient population

We investigated samples from CKD patients enrolled in a cross-sectional observational study. Patients were recruited from the outpatient clinics and departments of nephrology at Herlev Hospital and Rigshospitalet, University of Copenhagen, Denmark. The methodology and baseline data of this study have previously been reported in detail^61^. In brief, 81 patients with CKD were included on the basis of estimated glomerular filtration rate (eGFR) less than 90 mL/min/1.73 m^2^ (CKD stages 2–5) and age between 18 and 80 years. eGFR was calculated based on the Modification of Diet in Renal Disease (MDRD) formula^62^. Patients were divided into CKD stages according to eGFR, thus stage 2 represents eGFR 60–89 mL/min/1.73 m^2^, stage 3A: 45–59 mL/min/1.73 m^2^, stage 3B: 30–44 mL/min/1.73 m^2^,stage 4: 15–29 mL/min/1.73 m^2^) and stage 5: <15 mL/min/1.73 m^2^. Exclusion criteria were systolic blood pressure (BP) > 200 mmHg, renal replacement therapy, and ongoing use of immunosuppressive drugs. The cohort consisted of 20 patients with CKD stage 2, 21 patients with CKD stage 3, 22 patients with CKD stage 4, and 18 patients with CKD stage 5. All patients provided written informed consent, and the study was conducted in accordance with the Declaration of Helsinki. The study was approved by the national ethical committee (H-15002269; Center for Sundhed – De Videnskabsetiske Komiteer, Regionsgården, Kongens Vænge 2, 3400 Hillerød, Denmark).

### Sample description and handling

Patients collected 24 h urine the day leading up to the planned visit. Patients were fasted and samples were drawn before 12.00 (noon) and collected in EDTA-treated tubes. Samples were taken while the patient was seated, after which cells were removed from the plasma by centrifugation for 10 minutes at 1000 x g using a refrigerated centrifuge, aliquoted, and stored at −80 °C. Aliquots used for the current investigation have not been thawed prior to the study.

### Biomarkers

All of the utilized enzyme-linked immunosorbent serum assays (ELISAs) are listed in Table 2. The assays were measured according to manufacturer instructions (Nordic Bioscience), and all except sauC3M (sandwich ELISA) were competitive ELISAs. Each ELISA plate included kit control samples to monitor inter-assay variation. All samples were measured within the range of the specific assay. All samples below the lower limit of quantification (LLOQ) were assigned the value of LLOQ. Urinary biomarker levels were normalized for urinary creatinine concentration, measured with the QuantiChrom^TM^ Creatinine Assay Kit (Bioassay Systems, Hayward, USA). Samples analyzed for biomarkers in the present study were stored at −80° Celsius.

### Clinical and laboratory analyses

The Ambulatory BP measurement was made on the non-dominant arm by the oscillometric method using SpaceLabs 90217 equipment^61^. Cuff width was selected according to arm circumference. Recorders were programmed to obtain measurements every 15 minutes during daytime (8.00–23.00 hours) and every 30 minutes during the night (23.00–8.00 hours). A minimum of ten recordings during daytime and five during night time were required and a mean was calculated.

All routine blood and urine analyses were carried out at the Department of Clinical Chemistry at Herlev Hospital, Denmark, as previously described^61^. Blood samples were drawn at the examination day and analyzed for plasma (p) creatinine and p-cholesterol including p-LDL and p-HDL as well as p-triglycerides.

### Statistical analysis

Patients’ demographics, serological biomarkers and clinical characteristics are presented as medians with IQR for continuous variables, and number with percentages for categorical variables. Normality distribution was assessed by a D’Agostino-Pearson omnibus normality test. There were no missing values for clinical characteristics or biomarkers in the study.

CKD heterogeneity was assessed using principal component followed by unsupervised cluster analysis. Principal component analysis (PCA) was performed using log-transformed biomarkers in order to identify the dimensions with highest information which were subsequently used for clustering. Based on the PCA results for each patient, unsupervised clustering with the Ward method and Euclidian distance was made. The number of appropriate clusters was determined using the metrics provided in the NbClust package in R^63^. Clinical characteristics were not included in the cluster analyses and were used to characterize the clusters.

To test for differences between clusters, ANOVA was used on normally distributed data and a Kruskal-Wallis test was used on non-normally distributed continuous data. These analyses were performed with non-transformed biomarkers. Differences in categorical variables were determined by a Chi-Squared test. In the case of multiple comparisons, we utilized Dunn’s multiple comparison post-hoc test for the Kruskal-Wallis test.

PCA and clustering were performed with the R project software^63,64^. Statistical analyses to compare cluster were made by MedCalc (MedCalc, Belgium) and GraphPad Prism, version 6 (GraphPad Software, San Diego, CA, USA). All two-sided p-values below 0.05 were considered significant.

## Supplementary information

Supplemental Table 1. Biomarker levels in patients stratified by CKD stage

## Data Availability

The datasets generated and analysed during the current study are available from the corresponding author on reasonable request.

## References

[1] Mills KT (2015). A systematic analysis of worldwide population-based data on the global burden of chronic kidney disease in 2010. Kidney Int..

[2] Jha V (2013). Chronic kidney disease: global dimension and perspectives. Lancet.

[3] Hill, N. *et al*. Prevalence of Chronic Kidney Disease - A Systematic Review and Meta-Analysis. *PLoS One* **11** (2016). 10.1371/journal.pone.0158765 PMC4934905 27383068

[4] Risdon RA, Sloper JC, De Wardener HE (1968). Relationship Between Renal Function and Histological Changes Found in Renal-Biopsy Specimens From Patients With Persistent Glomerular Nephritis. Lancet.

[5] Schainuk LI, Striker GE, Cutler RE, Benditt EP (1970). Structural-functional correlations in renal disease. Hum. Pathol..

[6] Bohle A, Mackensen-Haen S, von Gise H (1987). Significance of tubulointerstitial changes in the renal cortex for the excretory function and concentration ability of the kidney: a morphometric contribution. Am. J. Nephrol..

[7] Kuncio GS, Neilson EG, Haverty T (1991). Mechanisms of tubulointerstitial fibrosis. Kidney Int..

[8] Harris RC, Neilson EG (2006). Toward a unified theory of renal progression. Annu. Rev. Med..

[9] Liu Y (2011). Cellular and molecular mechanisms of renal fibrosis. Nat Rev Nephrol..

[10] Genovese F, Manresa AA, Leeming DJ, Karsdal MA, Boor P (2014). The extracellular matrix in the kidney: a source of novel non-invasive biomarkers of kidney fibrosis?. Fibrogenesis Tissue Repair.

[11] Cescon M, Gattazzo F, Chen P, Bonaldo P (2015). Collagen VI at a glance. J. Cell Sci..

[12] Catania JM, Chen G, Parrish AR (2007). Role of matrix metalloproteinases in renal pathophysiologies. Am. J. Physiol. Renal Physiol..

[13] Cheng Z (2017). MMP-2 and 9 in Chronic Kidney Disease. Int. J. Mol. Sci..

[14] Mayer U (1994). Recombinant expression and properties of the Kunitz-type protease-inhibitor module from human type VI collagen a3 (VI) chain. Eur. J. Biochem..

[15] Aigner T, Hambach L, So S, Schlo U (2002). The C5 Domain of Col6A3 Is Cleaved Off from the Col6 Fibrils Immediately after Secretion. Biochem. Biophys. Res. Commun..

[16] Leeming DJ (2012). Enzyme-linked immunosorbent serum assay specific for the 7S domain of collagen type IV (P4NP 7S): A marker related to the extracellular matrix remodeling during liver fibrogenesis. Hepatol. Res..

[17] Leeming DJ, Karsdal MA, Rasmussen LM, Scholze A, Tepel M (2013). Association of Systemic Collagen Type IV Formation with Survival among Patients Undergoing Hemodialysis. PLoS One.

[18] Sand JM (2013). MMP mediated degradation of type IV collagen alpha 1 and alpha 3 chains reflects basement membrane remodeling in experimental and clinical fibrosis - Validation of two novel biomarker assays. PLoS One.

[19] Nielsen MJ (2013). The neo-epitope specific PRO-C3 ELISA measures true formation of type III collagen associated with liver and muscle parameters. Am J Transl Res.

[20] Nielsen M (2014). Plasma Pro-C3 (N-terminal type III collagen propeptide) predicts fibrosis progression in patients with chronic hepatitis C. Liver Int..

[21] Jansen C (2014). PRO-C3-Levels in patients with HIV/HCV-Co-Infection reflect fibrosis stage and degree of portal hypertension. PLoS One.

[22] Rasmussen, D. G. K., Sand, J. M. B., Karsdal, M. A. & Genovese, F. Development of a Novel Enzyme-Linked Immunosorbent Assay Targeting a Neo- Epitope Generated by Cathepsin-Mediated Turnover of Type III Collagen and Its Application in Chronic Obstructive Pulmonary Disease. *PLoS One*, 1–14, 10.1371/journal.pone.0170023 (2017). 10.1371/journal.pone.0170023 PMC5226775 28076408

[23] Barascuk N (2010). A novel assay for extracellular matrix remodeling associated with liver fibrosis: An enzyme-linked immunosorbent assay (ELISA) for a MMP-9 proteolytically revealed neo-epitope of type III collagen. Clin. Biochem..

[24] Papasotiriou M (2015). Serum and urine markers of collagen degradation reflect renal fibrosis in experimental kidney diseases. Nephrol Dial Transpl..

[25] Genovese F (2016). Turnover of type III collagen reflects disease severity and is associated with progression and microinflammation in patients with IgA nephropathy. Nephrol Dial Transpl..

[26] Leeming DJ (2010). Enzyme-linked immunosorbent serum assays (ELISAs) for rat and human N-terminal pro-peptide of collagen type I (PINP) - Assessment of corresponding epitopes. Clin. Biochem..

[27] Leeming D (2011). A novel marker for assessment of liver matrix remodeling: An enzyme-linked immunosorbent assay (ELISA) detecting a MMP generated type I collagen neo-epitope (C1M). Biomarkers.

[28] Sun S (2015). Collagen type III and VI turnover in response to long-term immobilization. PLoS One.

[29] Fenton A (2017). Serum endotrophin, a type VI collagen cleavage product, is associated with increased mortality in chronic kidney disease. PLoS One.

[30] Rasmussen DGK (2017). Urinary endotrophin predicts disease progression in patients with chronic kidney disease. Sci. Rep..

[31] Rasmussen DGK (2018). Higher collagen VI formation is associated with all-cause mortality in patients with type 2 diabetes and microalbuminuria. Diabetes Care.

[32] Pilemann-Lyberg S (2019). Markers of Collagen Formation and Degradation Reflect Renal Function and Predict Adverse Outcomes in Patients With Type 1 Diabetes. Diabetes Care.

[33] Veidal SS (2011). MMP mediated degradation of type VI collagen is highly associated with liver Fibrosis - Identification and validation of a novel biochemical marker assay. PLoS One.

[34] Sand JMB (2016). High levels of biomarkers of collagen remodeling are associated with increased mortality in COPD - results from the ECLIPSE study. Respir. Res..

[35] Nielsen MJ (2015). Plasma Pro-C3 (N-terminal type III collagen propeptide) predicts fibrosis progression in patients with chronic hepatitis C. Liver Int..

[36] Karsdal, M. A. *et al*. Novel insights into the function and dynamics of extracellular matrix in liver fibrosis. *Am*. *J*. *Physiol*. *- Gastrointest*. *Liver Physiol*. **308** (2015). 10.1152/ajpgi.00447.2014 PMC4437019 25767261

[37] Nerlich AG, Schleicher ED, Wiest I, Speckes U, Timpl R (1994). Immunohistochemical localization of collagen VI in diabetic glomeruli. Kidney Int..

[38] Groma V (1998). Demonstration of collagen type VI and alpha-smooth muscle actin in renal fibrotic injury in man. Nephrol Dial Transpl..

[39] Adair-Kirk TL, Senior RM (2008). Fragments of Extracellular Matrix as Mediators of Inflammation. Int J Biochem Cell Biol..

[40] Arroyo AG, Iruela-Arispe ML (2010). Extracellular matrix, inflammation, and the angiogenic response. Cardiovasc. Res..

[41] Magro G, Grasso S, Colombatti A, Lopes M (1996). Immunohistochemical distribution of type VI collagen in developing human kidney. Histochem. J..

[42] Zhu D (1994). Application of electron microscopic immunocytochemistry to the human kidney: Distribution of type IV and type VI collagen in normal human kidney. J. Histochem. Cytochem..

[43] Kuo HJ, Maslen CL, Keene DR, Glanville RW, Type VI (1997). collagen anchors endothelial basement membranes by interacting with type IV collagen. J. Biol. Chem..

[44] Bober M, Enochsson C, Collin M, Mörgelin M (2010). Collagen VI is a subepithelial adhesive target for human respiratory tract pathogens. J. Innate Immun..

[45] Dassah MA (2014). Annexin A2 mediates secretion of collagen VI, pulmonary elasticity and apoptosis of bronchial epithelial cells. J. Cell Sci..

[46] Izu Y (2011). Dysfunctional tendon collagen fibrillogenesis in collagen VI null mice. Matrix Biol..

[47] Iyengar P (2005). Adipocyte-derived collagen VI affects early mammary tumor progression *in vivo*, demonstrating a critical interaction in the tumor/stroma microenvironment. J. Clin. Invest..

[48] Cattaruzza S (2013). NG2/CSPG4-collagen type VI interplays putatively involved in the microenvironmental control of tumour engraftment and local expansion. J. Mol. Cell Biol..

[49] Park J, Scherer PE (2012). Adipocyte-derived endotrophin promotes malignant tumor progression. J. Clin. Invest..

[50] Dobrin PB, Baker WH, Gley WC (1984). Elastolytic and Collagenolytic Studies of Arteries: Implications for the Mechanical Properties of Aneurysms. Arch. Surg..

[51] Dobrin PB, Mrkvicka R (1994). Failure of elastin or collagen as possible critical connective tissue alterations underlying aneurysmal dilatation. Cardiovasc. Surg..

[52] Henkel W, Glanville RW (1982). Covalent crosslinking between molecules of type I and type III collagen. The involvement of the N-terminal, nonhelical regions of the alpha 1 (I) and alpha 1 (III) chains in the formation of intermolecular crosslinks. Eur. J. Biochem..

[53] Asgari, M., Latifi, N., Heris, H. K., Vali, H. & Mongeau, L. *In vitro* fibrillogenesis of tropocollagen type III in collagen type i affects its relative fibrillar topology and mechanics. *Sci*. *Rep*. **7** (2017). 10.1038/s41598-017-01476-y PMC5431193 28469139

[54] Merkel JR, DiPaolo BR, Hallock GG, Rice DC, Type I (1988). and type III collagen content of healing wounds in fetal and adult rats. Proc. Soc. Exp. Biol. Med..

[55] Vleming LJ (1995). Progression of chronic renal disease in humans is associated with the deposition of basement membrane components and decorin in the interstitial extracellular matrix. Clin. Nephrol..

[56] Good DM (2010). Naturally Occurring Human Urinary Peptides for Use in Diagnosis of Chronic Kidney Disease. Mol. Cell. Proteomics.

[57] Argilés, À. *et al*. CKD273, a New Proteomics Classifier Assessing CKD and Its Prognosis. *PLoS One* **8** (2013). 10.1371/journal.pone.0062837 PMC3653906 23690958

[58] Siwy J (2014). Multicentre prospective validation of a urinary peptidome-based classifier for the diagnosis of type 2 diabetic nephropathy. Nephrol. Dial. Transplant.

[59] Rossing K (2008). Urinary proteomics in diabetes and CKD. J. Am. Soc. Nephrol..

[60] Øvrehus MA, Zürbig P, Vikse BE, Hallan SI (2015). Urinary proteomics in chronic kidney disease: diagnosis and risk of progression beyond albuminuria. Clin. Proteomics.

[61] Boesby L, Thijs L, Elung-Jensen T, Strandgaard S, Kamper A (2012). Ambulatory arterial stiffness index in chronic kidney disease stage 2-5. Reproducibility and relationship with pulse wave parameters and kidney function. Scand. J. Clin. Lab. Invest..

[62] Levey AS (1999). A more accurate method to estimate glomerular filtration rate from serum creatinine: a new prediction equation. Modification of Diet in Renal Disease Study Group. Ann. Intern. Med..

[63] Charrad M, Ghazzali N, Boiteau V, Niknafs A (2014). NbClust: An R Package for Determining the Relevant Number of Clusters in a Data Set. J. Stat. Softw..

[64] R Core Team. R: A Language and Environment for Statistical Computing (2016).

