# Supplementary material for: Collagen turnover profiles in chronic kidney disease
Source: Sci Rep. 2019 Nov 5;9:16062. doi: 10.1038/s41598-019-51905-3 (PMC6831687; doi:10.1038/s41598-019-51905-3)
Supplement: Supplementary file 1 — Supplemental Table 1. Biomarker levels in patients stratified by CKD stage [file 41598_2019_51905_MOESM1_ESM.doc]

**Collagen turnover profiles in chronic kidney disease**

Daniel Guldager Kring Rasmussen^1,2^, PhD, Lene Boesby^3,4^, MD, Signe Holm Nielsen^1,5^, PhD, Martin Tepel^2,6^, MD, Sophie Birot^1^, PhD, Morten Asser Karsdal^1^, PhD, Anne-Lise Kamper^7^, MD, Federica Genovese^1^, PhD

**Supplemental Table 1. Biomarker levels in patients stratified by CKD stage**

| **Degradation markers** | | | | | |
| --- | --- | --- | --- | --- | --- |
|  | **Stage 2 (n=20)** | **Stage 3 (n=21)** | **Stage 4 (n=22)** | **Stage 5 (n=18)** | **p-value** |
| **P-C1M^b^** | 12.3 [10.9-13.9] | 13.4 [11.5-15.3] | 12.2 [11.2-13.3] | 12.1 [10.7-13.7] | 0.92 |
| **U-C1M^b^** | 9.2 [7.7-10.7] | 10.1 [8.3-12.3] | 10.3 [8.6-11.9] | 12.0 [8.7-16.6] | 0.41 |
| **P-C3A^a^** | 58.4 [54.6-62.2] | 58.8 [53.7-63.9] | 61.2 [57.1-65.3] | 57.0 [53.0-60.9] | 0.55 |
| **P-C3C^a^** | 2.8 [2.1-3.6] | 3.2 [2.8-3.6] | 3.0 [2.6-3.4] | 3.2 [2.7-3.6] | 0.70 |
| **U-C3C ^b^** | 11.2 [9.2-13.5] | 11.3 [9.1-13.9] | 13.2 [10.3-16.1] | 13.7 [9.5-19.8] | 0.90 |
| **P-C3M^a^** | 10.6 [10-11.3] | 11.4 [10.4-13.4] | 10.3 [9.0-11.7] | 9.8 [8.8-10.8] | 0.18 |
| **U-C3M ^b^** | 28.7 [23.5-34.0] | 20.1 [17.1-23.1] | 10.1 [8.5-11.9] | 10.6 [6.8-16.4] | **<0.001** |
| **U-sauC3M ^b^** | 2.7 [2.2-3.3] | 1.8 [1.5-2.2] | 0.9 [0.7-1.1] | 0.5 [0.5-0.6] | **<0.001** |
| **P-C4M^a^** | 25.5 [22.5-28.4] | 26.5 [24.2-28.7] | 25.3 [22.6-28.0] | 24.8 [22.9-26.7] | 0.81 |
| **U-C4M ^b^** | 10.4 [8.7-12.0] | 10.9 [9.1-13.1] | 10.2 [9.2-11.2] | 10.3 [7.5-14.1] | 0.52 |
| **P-C4Ma3^b^** | 2.2 [1.8-2.7] | 2.4 [2.0-2.9] | 2.0 [1.6-2.3] | 1.7 [1.4-2.1] | 0.12 |
| **P-PRO-C4^a^** | 208.8 [192.3-225.4] | 226.4 [202.0-250.8] | 198.1 [172.5-223.7] | 168.5 [148.7-188.4] | **0.004** |
| **P-C6M^b^** | 5.0 [4.0-5.9] | 5.8 [4.9-7.0] | 4.9 [3.8-6.4] | 4.9 [3.6-6.1] | 0.23 |
| **Formation markers** | | | | | |
|  | **Stage 2 (n=20)** | **Stage 3 (n=21)** | **Stage 4 (n=22)** | **Stage 5 (n=18)** | **p-value** |
| **P-PRO-C1^a^** | 105.0 [81.7-128.3] | 107.9 [79.9-135.8] | 125.6 [97.9-153.3] | 153.5 [106.3-200.7] | 0.12 |
| **P-PRO-C3^b^** | 16.3 [12.9-20.5] | 18.8 [16.2-21.4] | 12.2 [15.9-20.8] | 19.6 [14.9-25.7] | 0.13 |
| **U-PRO-C3 ^b^** | 5.1 [3.6-7.3] | 5.7 [4.6-7.0] | 5.8 [4.6-7.3] | 8.8 [4.9-15.7] | **0.03** |
| **P-PRO-C6^b^** | 10.7 [9.4-12.1] | 14.0 [12.2-15.9] | 25.9 [23.0-28.9] | 37.6 [33.4-42.5] | **<0.001** |
| **U-PRO-C6 ^b^** | 3.4 [2.0-2.9] | 3.0 [2.1-4.4] | 15.6 [8.0-30.3] | 77.2 [41.0-145.4] | **<0.001** |

Data is presented as ^a^mean or ^b^median with 95% confidence interval. ^a^Normally distributed data, or data where log-transformation led to a normal distribution, was assessed for significance using ANOVA. ^b^Non-normally distributed data was assessed for significance using a Kruskal-Wallis test.
